# Case of Painful Affection of the Bladder, from Gastric and Hepatic Disorder

**Published:** 1818-10-01

**Authors:** 


### VIII.

### *Case of painful Affection of the Bladder, from Gastric and Hepatic Disorder.*

By a Physician of London.

IN the year 1807, I was consulted by an old gentleman who had often laboured, during several years previously, under what was deemed by his medical attendant, a spasmodic affection of the bladder. The attack was generally ushered in by some degree of fever, attended with frequent and severe, but not permanent pains in the region of the bladder; and there was either a retention of urine, or it was passed in small quantity, and with considerable difficulty. He had tried a great variety of measures at different periods, but had only found a palliative relief from the best; for the disease still returned, at intervals, with as much violence as before, and indeed, on most occasions, he appeared to obtain alleviation rather from the efforts of nature than of art; a spontaneous diarrhœa at last generally mitigating or removing the urgent symptoms for a time. When I first saw him, he was suffering under an attack of unusual duration and severity, since it had continued for upwards of six weeks, with only slight intermissions of perfect ease. On examining into the character of the case, I found that the tongue was exceedingly foul; the stomach flatulent, and the biliary secretion much disordered, the stools having nearly the colour and consistence of tar, and the urine being scanty, high coloured, and somewhat tinged with bile. Besides, his spirits were extremely oppressed at some periods, and equally variable at others; and he had that nervous irritability about him which is so often a conspicuous attribute of hepatic disturbance. As he passed his urine well at intervals, and as the whole history of the case seemed to indicate that there was no fixed and progressive disease in the bladder itself, and as a spontaneous looseness mostly abated the symptoms, it struck me that the frequent seizures of pain referred to the bladder, might all along, perhaps, have been dependent upon the state of the stomach, liver, and intestines. Under this impression, I ordered the patient a regular course of purgative medicines, exhibiting calomel and jalap almost every night, and some neutral salts to determine their action on the bowels, on the subsequent morning. This plan was continued till the mouth became slightly affected, the stools natural, and the tongue clean; but as he had been accustomed to live generously, and as he was of an advanced age, a light nourishing diet, with moderate portions of wine and malt liquor, were allowed him, with a view of supporting his strength under the evacuant and alterative treatment. During the first week of this plan, the attacks of pain in the bladder became less frequent and severe, and in less than a month they disappeared; though it ought to be mentioned, that he used the warm bath occasionally. In fact, he had no violent fits of uneasiness afterwards; but whenever his bowels became torpid or irregular, he had some threatenings of his former complaint, which, however, were always speedily removed by a repetition of the brisk purgatives. He passed some years of tolerably good health, and at last died of an affection of the heart, complicated with abdominal disease. But as far as the painful affection of the bladder was concerned in this example, the previous brief history may serve to shew, that a disease is not necessarily seated in the organ where the pain is seated, but that its latent cause may be in another part; and this particular instance of sympathy is the more valuable in a practical view, because I have met with some similar cases in old persons where the pain in the bladder had misled other practitioners as to the real nature and source of the disease. The investigation of morbid sympathies is a subject of the highest importance in medical science; and when it is better understood by the fair detail and induction of facts, many new lights will be thrown upon pathology and practice.

*September* 1, 1818.

